# SakA and MpkC Stress MAPKs Show Opposite and Common Functions During Stress Responses and Development in *Aspergillus nidulans*

**DOI:** 10.3389/fmicb.2018.02518

**Published:** 2018-10-23

**Authors:** Verónica Garrido-Bazán, Rafael Jaimes-Arroyo, Olivia Sánchez, Fernando Lara-Rojas, Jesús Aguirre

**Affiliations:** ^1^Departamento de Biología Celular y del Desarrollo, Instituto de Fisiología Celular, Universidad Nacional Autónoma de México, Mexico City, Mexico; ^2^Posgrado en Ciencias Biológicas, Unidad de Posgrado, Mexico City, Mexico

**Keywords:** oxidative stress, cell-wall stress, spore germination, spore development, spore viability, MAPK nuclear localization, MAPKK

## Abstract

Stress activated MAP kinases (SAPKs) of the Hog1/Sty1/p38 family are specialized in transducing stress signals. In contrast to what is seen in animal cells, very few fungal species contain more than one SAPK. *Aspergillus nidulans* and other Aspergilli contain two SAPKs called SakA/HogA and MpkC. We have shown that SakA is essential for conidia to maintain their viability and to survive high H_2_O_2_ concentrations. H_2_O_2_ induces SakA nuclear accumulation and its interaction with transcription factor AtfA. Although SakA and MpkC show physical interaction, little is known about MpkC functions. Here we show that *ΔmpkC* mutants are not sensitive to oxidative stress but in fact MpkC inactivation partially restores the oxidative stress resistance of *ΔsakA* mutants. *ΔmpkC* mutants display about twofold increase in the production of fully viable conidia. The inactivation of the SakA upstream MAPKK PbsB or the simultaneous elimination of *sakA* and *mpkC* result in virtually identical phenotypes, including decreased radial growth, a drastic reduction of conidiation and a sharp, progressive loss of conidial viability. SakA and to a minor extent MpkC also regulate cell-wall integrity. Given the roles of MpkC in conidiation and oxidative stress sensitivity, we used a functional MpkC::GFP fusion to determine MpkC nuclear localization as an *in vivo* indicator of MpkC activation during asexual development and stress. MpkC is mostly localized in the cytoplasm of intact conidia, accumulates in nuclei during the first 2 h of germination and then becomes progressively excluded from nuclei in growing hyphae. In the conidiophore, MpkC nuclear accumulation increases in vesicles, metulae and phialides and decreases in older conidia. Oxidative and osmotic stresses induce MpkC nuclear accumulation in both germinating conidia and hyphae. In all these cases, MpkC nuclear accumulation is largely dependent on the MAPKK PbsB. Our results indicate that SakA and MpkC play major, distinct and sometimes opposing roles in conidiation and conidiospore physiology, as well as common roles in response to stress. We propose that two SAPKs are necessary to delay (MpkC) or fully stop (SakA) mitosis during conidiogenesis and the terminal differentiation of conidia, in the highly prolific phialoconidiation process characteristic of the Aspergilli.

## Introduction

Eukaryotic cells use MAP kinase cascades, composed of a MAPK, a MAPK kinase (MAPKK), and a MAPKK kinase (MAPKKK), to transmit environmental signals. Active phosphorylated MAPKs phosphorylate multiple targets, including other enzymes and usually translocate from cytoplasm to nucleus to phosphorylate nuclear targets such as transcription factors. In contrast, upstream MAPKK and MAPKKK phosphorylate only the immediate downstream kinase in the cascade. The topology of this basic module is enough to achieve transient, sustained and oscillatory responses. However, input/response dynamics is greatly affected by the presence of negative/positive feedback loops, scaffolding proteins and spatial gradients of kinases and phosphatases. Moreover, the range of responses is further expanded by the presence of several isoforms (i.e., p38 in animal cells) of a specific type of kinase (see Kholodenko and Birtwistle, 2009 for a review).

Classical stress-activated protein kinases or SAPKs are MAP kinases specialized in transducing multiple stress signals. In fungi, SAPK input typically involves a phosphorelay signal transduction system. Other MAP kinases, members of the MpkA and MpkB families, transduce specific signals such as cell-wall stress and hormone signals, through membrane sensors or G-protein coupled receptors (Grice et al., 2013). *Saccharomyces cerevisiae* Hog1, the first SAPK identified (Brewster et al., 1993) has been studied in great detail mainly as a pathway connected to osmoresistance and cell-cycle regulation (Escote et al., 2004). Likewise, *Schizosaccharomyces pombe* Sty1/Spc1 has been extensively characterized as a multi-stress responding SAPK involved in stress resistance and in cell-cycle control, mainly through the MAP kinase-activated protein kinase (MAPKAP) Srk1 (Lopez-Aviles et al., 2008; Shiozaki, 2009; Smith et al., 2010).

In filamentous fungi, *Magnaporthe grisea* OSM1, was the first HOG1/Spc1/p38 homolog studied, and shown to be required for normal asexual sporulation (mutants producing about 10 times less conidia), osmoresistance and arabitol biosynthesis (Dixon et al., 1999). Later, two independent groups cloned the *Aspergillus nidulans* HOG1 homolog and named it *hogA* (Han and Prade, 2002) and *sakA* (Kawasaki et al., 2002). Han and Prade (2002), reported that *hogA* expression was transiently induced by high osmolarity and that *ΔhogA* mutants showed decreased growth in the presence of 1–1.5 M NaCl at low (30°C) but not at *A. nidulans* normal growth temperature (37°C). Kawasaki et al. (2002), reported that SakA was transiently phosphorylated in response to both osmotic and oxidative stress, as well as early after the induction of asexual sporulation (conidiation), and that while *ΔsakA* mutants were not sensitive to osmotic stress, they produced asexual spores that progressively lost their viability and were sensitive to oxidative and heat shock stress (Kawasaki et al., 2002). In most fungi the constitutive activation of the SAPK pathway results in lethality and in fact, this is the action mechanism of common fungicides such as fludioxonil. In filamentous fungi in which the HOG1 pathway is solely responsible for providing resistance to osmotic stress, its elimination is enough to confer resistance to fludioxonil. In contrast, in *A. nidulans* and other filamentous fungi (Izumitsu et al., 2007) osmoresistance is regulated by both SakA and response regulator SrrA, and it is necessary to eliminate either the common upstream histidine kinase NikA or both, SakA and SrrA, to produce osmosensitivity (Vargas-Perez et al., 2007).

In *A. nidulans*, SakA is also phosphorylated in response to nutrient starvation and hypoxia stress (Lara-Rojas et al., 2011), and it mediates light responses (Fischer et al., 2016; Yu et al., 2016). When phosphorylated, it translocates to nuclei, where it physically interacts with transcription factor AtfA, to regulate the expression of several genes in response to oxidative (Lara-Rojas et al., 2011) and osmotic stress (Hagiwara et al., 2009). SakA also interacts with the MAPKAP SrkA, a homolog of *S. pombe* Srk1, and mediates its nuclear localization in response to oxidative stress. Also in response to H_2_O_2_, SakA interacts with several other proteins, some related to cell-cycle regulation (Jaimes-Arroyo et al., 2015).

SakA also links stress environmental sensing and development, playing essential roles in the transition between growth and differentiation. *ΔsakA* mutants show a strong de-repression of NADPH oxidase gene *noxA*, essential for sexual development (Lara-Ortiz et al., 2003), and a highly exacerbated sexual development (Kawasaki et al., 2002). During asexual development, *ΔsakA* intact conidia progressively lose their viability and this is consistent with the developmental phosphorylation and nuclear accumulation of SakA in intact conidia. Moreover, SakA needs to be dephosphorylated for germination of conidia to take place (Lara-Rojas et al., 2011). In many other fungi where the single SakA ortholog present has been studied, it has been linked to stress sensing and the regulation of development or pathogenicity (Segmuller et al., 2007; Lamb et al., 2012; Nimmanee et al., 2015; Esquivel-Naranjo et al., 2016).

The presence of more than one SAPK in a single fungal species was first documented in *A. nidulans*, where the *mpkC* gene was identified (GenBank accession numbers: AF195773 and AN4668) and the protein compared to SakA (Kawasaki et al., 2002). SakA (379 amino-acids) and MpkC (415 amino-acids) are 62% identical, both being substrates of the upstream MAPKK PbsB (Furukawa et al., 2005) and showing physical interaction (Jaimes-Arroyo et al., 2015). Unexpectedly, the deletion *mpkC* did not generate any clear phenotype (Jun et al., 2011).

More recently, two SAPKs have been reported in the obligatory halophilic basidiomycetous *Wallemia ichthyophaga* and the ascomycetous yeast-like fungus *Hortaea werneckii*. Having virtually the same size and being 69% identical, *W. ichthyophaga* WiHog1A and WiHog1B genes are differentially induced by high osmolarity and show different degrees of complementation of a *S. cerevisiae Δhog1* mutant (Konte and Plemenitas, 2013). With evidence supporting an ancestral duplication of its entire genome, *H. werneckii* contains the two nearly identical (95%) and functionally redundant SAPKs HwHog1A and HwHog1B, which show osmolyte-type-dependent phosphorylation (Kejzar et al., 2015).

To evaluate the relative contribution of SakA and MpkC in stress sensing and development, we decided to characterized single and double *ΔmpkC* and *ΔsakA* null mutants and compare them with mutants in which the upstream MAPKK gene *pbsB* was deleted. In addition, we studied the nuclear localization of a functional MpkC::GFP fusion during stress and development in wild type and *ΔpbsB* genetic backgrounds, as a visual tool to detect MpkC activation *in vivo*.

## Materials and Methods

### Strains, Media, and Growth Conditions

**FIGURE 1:**
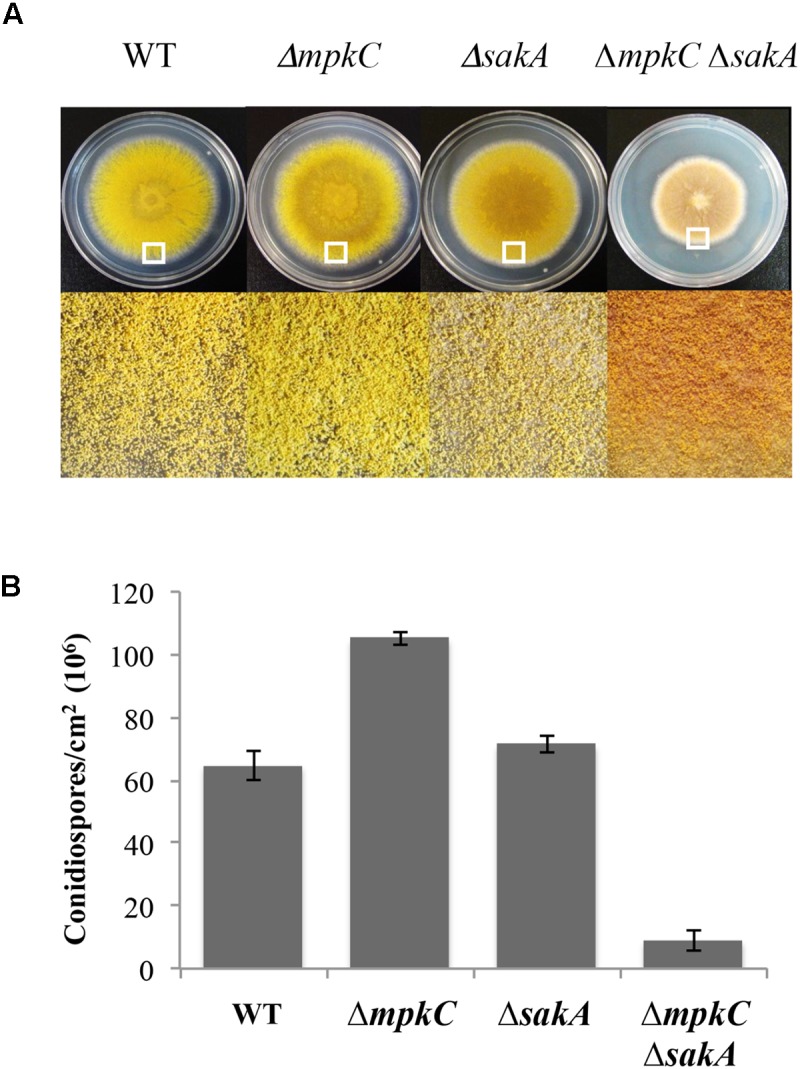
*sakA* and *mpkC* deletion show opposing effects on conidiation but both genes are needed for normal conidiation. **(A)** Asexual spores (1 × 10^4^) from strains CLK43 (WT), CFL10 (*ΔmpkC*), TOL1 (*ΔsakA*) and CFL12 (*ΔmpkC ΔsakA*) were inoculated on supplemented MM plates and incubated at 37°C during 5 days. **(B)** Total conidiospores per colony were harvested, counted, and the count divided by the colony area to obtain the number of conidiospores per square centimeter. Bars indicate standard deviation from three independent experiments. A representative experiment is shown. The white squares in panel **(A)** indicate the colony regions enlarged at the bottom of the figure. See Supplementary Table S1 for strain full genotypes.

*Aspergillus nidulans* strains used in this work are listed in Supplementary Table S1 (McCluskey et al., 2010). All strains were grown at 37°C in glucose minimal nitrate medium (Hill and Käfer, 2001), plus supplements. H_2_O_2_ was added to agar medium at ∼50°C before solidification. H_2_O_2_-containing plates were used the day they were prepared or stored at 4°C for no more than 24 h. 6 cm diameter plates were used in solid media experiments, except in Figure 1, where we used 10 cm plates. For mycelial stress sensitivity assays, mycelial plugs of the same area (diameter, 0.5 cm) were cut from the growing edge of 5-day colonies using a cork borer. Agar excess was removed and the mycelial mat was transferred to the testing medium.

The different gene-deletion constructs were produced by double joint PCR (Yu et al., 2004) using genomic DNA as template and different primer combinations. Primers are listed in Supplementary Table S2. To delete the *mpkC* gene (AN4668), PCR fragments were generated with primers 5′For-mpkC/5′Rev-mpkC and 3′For-mpkC/3′Rev-mpkC. *Aspergillus fumigatus pyrG* marker was amplified with primers pyrGforward and pyrGreverse, using plasmid PFNO3 as template (Nayak et al., 2006). These three fragments were purified, mixed and used in a fusion PCR with primers 5′Nest-mpkC and 3′Nest-mpkC. The final 4300 bp mpkC–AfpyrG–mpkC cassette was purified and used to transform *A. nidulans* strain MH11035 by electroporation (Sánchez and Aguirre, 1996; Sánchez et al., 1998). One PyrG+ transformant was obtained, analyzed by Southern blot to confirm *mpkC* elimination and named TFL8. TFL8 was crossed with strain CFL3 to remove the Δ*nkuA* mutation, and progeny strains CFL8 and CFL10 were confirmed by PCR and used in further experiments. To obtain *ΔmpkC* strain COS0020*ΔmpkC*, strains TFL8 and CLK43 were crossed to remove the *nkuA* deletion. COS0020*ΔmpkC* was confirmed by PCR and used in further experiments. To obtain *ΔsakA ΔmpkC* double mutants, strains TFLΔsakA-03 and CFL8 were crossed and the progeny analyzed by PCR to confirm the presence of both gene deletions.

To delete *pbsB* gene (AN0931), genomic DNA was used as template to amplify *pbsB* fragments with primers pbsB5′Fw/pyrGpbsB5′Rv and pbsB3′pyrGFw/pbsB3′Rv. *A. fumigatus pyrG* marker was amplified with primers pyrGforward/pyrGreverse, as before. These three fragments were purified and mixed with primers RealNestedpbsB5′/RealNestedpbsBRev to produce a final 4307 bp pbsB–AfpyrG–pbsB cassette, which was then used to transform *A. nidulans* strain MH11035 by electroporation. Five PyrG+ transformants were obtained and analyzed by PCR to confirm the elimination of *pbsB*. Strain TOSΔpbsB03 was chosen and crossed to strain CLK43 to get rid of *nkuA* deletion. Progeny strain COSΔpbsB05 was confirmed by PCR and used in further experiments.

To constitutively express MpkC::GFP from the *gpdA* promoter, a PCR construct biA-pyroA-gpdA-mpkC-GFP-biA, was used to transform strain TFL22 (Jaimes-Arroyo et al., 2015). PyroA^+^ BiA^-^ transformants were analyzed for GFP signal and strain TRJ12 was selected for further experiments. The absence of mutations in TRJ12 *mpkC* ORF fused to GFP was confirmed by DNA sequencing. The MpkC::GFP construct was derived from a *pyroA* bearing plasmid containing the *gpdA* promoter fused to *mpkC* cDNA and GFP, cloned in the middle of the *biA* gene (Bayram et al., 2012). Strain TRJ12 was transformed with PCR construct gpdA-h2A-mrfp-phleo, which confers resistance to phleomycin and labels nuclei with Histone H2A fused to mRFP (Bayram et al., 2012), and transformant TRJ13 was chosen for further experiments.

The *gpdA(p)::h2A::mrfp* allele was introduced into a *ΔsakA* genetic background by crossing strains TRJ7 and CRJ1. The presence of labeled nuclei and *ΔsakA* deletion was confirmed by Epifluorescence microscopy and PCR, respectively, and strain CVG18 was selected. Crosses between strains CRJ11 × TRJ13 and CRJ11 × TRJ7 were carried out to introduce *biA::pyroA::gpdA(p)::mpkC::GFP::pyroA::biA* and/or *gpdA(p)::h2A::mrfp* alleles into *ΔmpkC*, *ΔsakA*, *or ΔmpkC ΔsakA* backgrounds. Selected progeny were first tested for the presence of mRFP and/or GFP signal using Epifluorescence microscopy, while the presence of *ΔmpkC* and/or *ΔsakA* deletions was confirmed by PCR. Strains CVG17 and CVG18 were selected for additional experiments. The same strategy was used to introduce *biA::pyroA::gpdA(p)::mpkC::GFP::pyroA::biA* and *gpdA(p)::h2A::mrfp* markers into a *ΔpbsB* background, starting by crossing strains TRJ7 or TRJ13 with strain COSΔpbsB05. Strains CVG10 and CVG20 were selected and the presence of *ΔpbsB* mutation was confirmed by PCR.

### Microscopy

Fluorescence microscopy images were captured *in vivo*. For MpkC::GFP detection during germination, conidia were germinated for 2, 4, or 7 h at 37°C and observed using confocal microscopy. For stress treatments, 6 h germinated conidia were treated or not with 10 mM H_2_O_2_ for 10 min and observed within the next 10 min, or were germinated for 6 h in the presence of 1.2 M sorbitol. Image processing and fluorescence quantification were made using Image J and ZEN 2012 (Carl Zeiss, Jena, Germany). To observe conidiophores, the growing edge of a MpkC::GFP colony grown for 3 days at 37°C was sectioned, a drop of water was added and the section was carefully covered with a coverslip. Different fields in which conidiophore structure was better preserved were chosen for observation using confocal microscopy. To observe growing hyphae, 14 h grown mycelia was treated or not with 10 mM H_2_O_2_ for 20 min or grown for 14 h in MM containing 1.2 M sorbitol and then observed using confocal microscopy. All images were acquired using a Zeiss LSM800 inverted laser scanning confocal microscope using a Plan-Apochromat 63×/1.4 oil immersion objective and 488 and 561 nm laser lines. Maximum intensity projections were obtained from Z-stack images collected every 15 μm through entire cell volume. Images were processed using software ZEN 2012 (Carl Zeiss, Jena, Germany).

## Results

### MAPKs SakA and MpkC Regulate Asexual Development and Radial Growth

**FIGURE 2:**
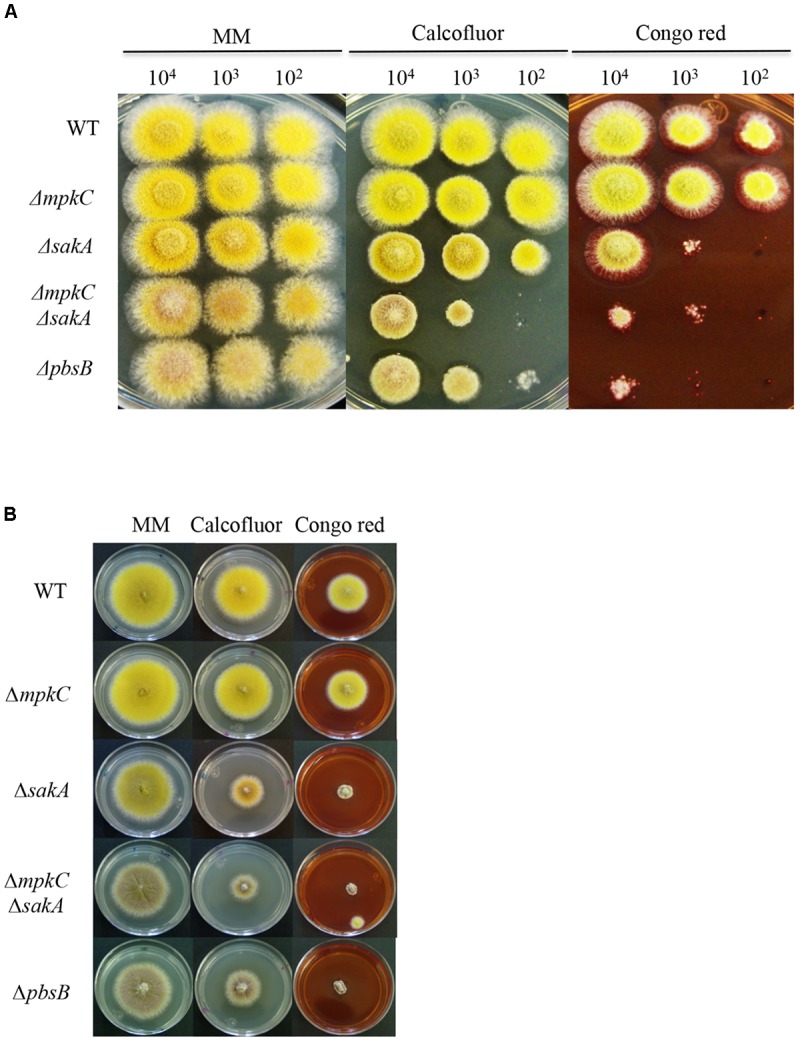
SakA and MpkC inactivation results in sensitivity to cell wall damaging in conidia and mycelia. **(A)** Spores (1 × 10^4^) from strains CLK43 (WT), COS0020ΔmpkC (*ΔmpkC*), CRJ1 (*ΔsakA*), CRJ11 (*ΔmpkC ΔsakA)* and COSΔpbsB05 (*ΔpbsB*) were inoculated on supplemented MM plates containing cell wall damaging compounds Calcofluor (20 μg/ml) or Congo red (30 μg/ml) and incubated at 37°C for 2 days. **(B)** Mycelial plugs cut from the growing edge of 5-day colonies from strains CLK43 (WT), COS0020ΔmpkC (*ΔmpkC*), CRJ1 (*ΔsakA*), CRJ11 (*ΔmpkC ΔsakA*), and COSΔpbsB05 (*ΔpbsB*) were transferred to plates containing Calcofluor (20 μg/ml) or Congo red (30 μg/ml) and incubated at 37°C for 4 days.

To compare SakA and MpkC functions in *A. nidulans*, we characterized mutants carrying deletions of *mpkC* and *sakA* genes. First, a *ΔmpkC* mutant was generated and single and double mutants were obtained by crosses (see section “Materials and Methods” and Supplementary Figure S1). In contrast to previous results showing that MpkC is necessary for polyalcohol sugar utilization in *A. fumigatus* (Reyes et al., 2006), we found that *ΔmpkC* and *ΔsakA* mutants grew as well as the wild type strain on different carbon sources, including mannitol and sorbitol (Supplementary Figure S2A). As reported before (Jun et al., 2011), *ΔmpkC* mutants did not display any obvious phenotype (Supplementary Figure S2B and Figure 2B). However, *ΔmpkC* colonies looked a little brighter, suggesting higher conidiation levels. To examine MpkC and SakA interactions during stress and conidiation, we generated a double *ΔmpkC ΔsakA* mutant and compared it with single *ΔmpkC* and *ΔsakA* mutants. While osmosentitivity was not increased by the simultaneous inactivation of MpkC and SakA (Supplementary Figure S2B), MpkC inactivation did affect conidiation. When compared to the wild type strain *ΔmpkC* mutants showed an increase in the production conidia, in sharp contrast with the *ΔmpkC ΔsakA* mutant, which presented a drastic reduction in conidiation as well as a clear reduction in radial growth (Figures 1A,B). While MpkC functions in conidiation were not evaluated in *A. fumigatus*, our results indicate that MpkC functions in polyalcohol sugar utilization are different in *A. fumigatus* (Reyes et al., 2006) and *A. nidulans*.

### MAPKK PbsB Is Required for the Function of Both SakA and MpkC

**FIGURE 3:**
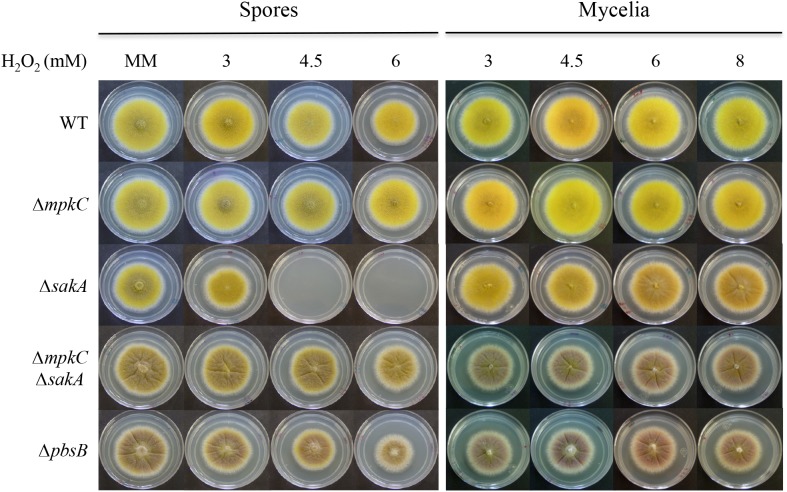
SakA and MpkC regulate conidia oxidative stress resistance in opposite ways. Conidia (1 × 10^4^) or mycelial plugs cut from the growing edge of 5-day colonies from strains CLK43 (WT), COS0020ΔmpkC (*ΔmpkC*), CRJ1 (*ΔsakA*), CRJ11 (*ΔmpkC ΔsakA*), and COSΔpbsB05 (*ΔpbsB*) were used to inoculate supplemented MM plates containing H_2_O_2_ at the indicated concentrations, and incubated at 37°C for 4 days.

To better understand the relationship between MpkC and SakA, we generated a mutant in which the upstream MAPKK gene *pbsB* was deleted (see section “Materials and Methods” and Supplementary Figure S3), as it has been shown that PbsB is necessary for SakA and MpkC phosphorylation (Furukawa et al., 2005). As shown in Figures 2, 3, *ΔpbsB* and *ΔmpkC ΔsakA* mutant growth and conidiation phenotypes were very similar, both producing similarly low amounts of conidia (Supplementary Figure S4A). Indeed, PbsB inactivation and the simultaneous inactivation of SakA and MpkC resulted in additional similar phenotypes (see further).

### SakA and PbsB Regulate Spore Viability, While SakA and MpkC Regulate Cell-Wall Integrity

Given that *ΔsakA* conidia suffer a progressive and dramatic loss of viability after they are formed (Kawasaki et al., 2002), we tested the viability of conidia produced by *ΔmpkC*, *ΔmpkC ΔsakA*, and *ΔpbsB* mutants. Results in Supplementary Figure S4B show that, like WT, *ΔmpkC* conidia remained fully viable for at least 40 days. In contrast, *ΔmpkC ΔsakA* conidia lost their viability at a much faster rate than conidia from the *ΔsakA* mutant. Consistent with this, conidia from the mutant lacking the MAPKK PbsB showed a viability loss rate similar to the one observed for *ΔmpkC ΔsakA* conidia.

In view of the growth reduction observed in the *ΔmpkC ΔsakA* and *ΔpbsB* mutants, we tested the role of these SAPKs in maintaining cell-wall integrity by plating conidia or mycelia from *ΔmpkC*, *ΔsakA*, *ΔmpkC ΔsakA*, and *ΔpbsB* mutants on media containing the cell-wall disturbing compounds calcofluor or Congo red. As shown in Figure 2A, the germination and growth of the *ΔmpkC* mutant was not affected by the presence of these compounds. In contrast, *ΔsakA* mutant growth was clearly reduced, particularly in the presence of Congo red. Moreover, *ΔmpkC ΔsakA* double mutant was even more sensitive to these compounds, clearly indicating that although SakA plays a more important role than MpkC, both SAPKs contribute to proper cell-wall biosynthesis. Again, *ΔmpkC ΔsakA* mutant phenotype was very similar to the one displayed by *ΔpbsB* mutant. When the same experiment was carried out using mycelia instead of conidia, *ΔsakA*, *ΔmpkC ΔsakA*, and *ΔpbsB* mutants showed similar sensitivity to calcofluor and Congo red suggesting that MpkC contribution to cell-wall integrity in mycelia is less important than in conidia (Figure 2B).

### MpkC and SakA Regulate Conidia Oxidative Stress Resistance in Opposite Ways

In contrast to their mycelial insensitivity to H_2_O_2_, *ΔsakA* mutants produce conidia that are sensitive to H_2_O_2_ (Kawasaki et al., 2002). To analyze MpkC contribution to this phenotype, we compared oxidative stress sensitivity of conidia and mycelia from *ΔmpkC*, *ΔsakA*, *ΔmpkC ΔsakA*, and *ΔpbsB* mutants. As shown in Figure 3, *ΔsakA* conidia were unable to grow at 4.5 and 6 mM H_2_O_2_, while *ΔmpkC* conidia showed wild type resistance to H_2_O_2_. Unexpectedly, MpkC inactivation restored the ability to *ΔsakA* mutants to grow at 4.5 and 6 mM H_2_O_2_ and a similar result was observed in the presence of tert-Butyl hydroperoxide (not shown). In contrast, a lack of *mpkC* or *pbsB* did not affect the sensitivity of mycelia to H_2_O_2_ (Figure 3). This indicates that SakA and MpkC regulate the response of intact conidia to oxidative stress in opposite ways. In line with this result, conidia from the *ΔpbsB* mutant, unable to activate both SakA and MpkC, were able to grow at 4.5 and 6 mM H_2_O_2_ (Figure 3). This indicates that PbsB is an upstream regulator of both SakA and MpkC, confirms the interactions observed in *ΔmpkC ΔsakA* mutants and show that in the absence of SakA, MpkC mediates a higher sensitivity of conidia to H_2_O_2_.

Overall, our results indicate that MpkC and SakA show complex interactions in opposing (conidiation and oxidative stress sensitivity), as well as concurrent (cell-wall biosynthesis) pathways, to regulate *A. nidulans* stress responses, growth, and development.

### Nuclear Localization of MpkC Is Developmentally Regulated in the Absence of External Stress, While Oxidative and Osmotic Stress Increase Its Nuclear Localization

Since *mpkC* gene is expressed at very low basal levels in *A. nidulans*, PbsB requirement for MpkC phosphorylation was shown expressing *mpkC* from an *A. oryzae* constitutive promoter (Furukawa et al., 2005). Recently, MpkC nuclear localization induced by osmotic stress was reported in *A. fumigatus* germinated conidia, using a MpkC::GFP fusion expressed form its native promoter (Bruder Nascimento et al., 2016). However, with such fusion GFP signal is virtually undetectable in the absence of stress. Here we decided to constitutively express MpkC tagged with GFP from the *gpdA* gene promoter, to examine the effects of MpkC expression and the role of PbsB in MpkC nuclear localization, under stressed and non-stressed conditions. To test that MpkC::GFP fusion was functional, we introduced it in WT, *ΔmpkC* and *ΔmpkC ΔsakA* backgrounds (Supplementary Figure S5). As shown in Supplementary Figures S6A,B, MpkC::GFP expression in a wild type background did not seem to affect growth, conidiation or stress sensitivity. In contrast, MpkC::GFP expression in *ΔmpkC* and *ΔmpkC ΔsakA* backgrounds was able to restore conidiation to wild type levels and to partially restore calcofluor and Congo red resistance of *ΔsakA* and *ΔmpkC ΔsakA* mutants (Supplementary Figure S6C). These results indicated that this MpkC::GFP fusion was functional.

**FIGURE 4:**
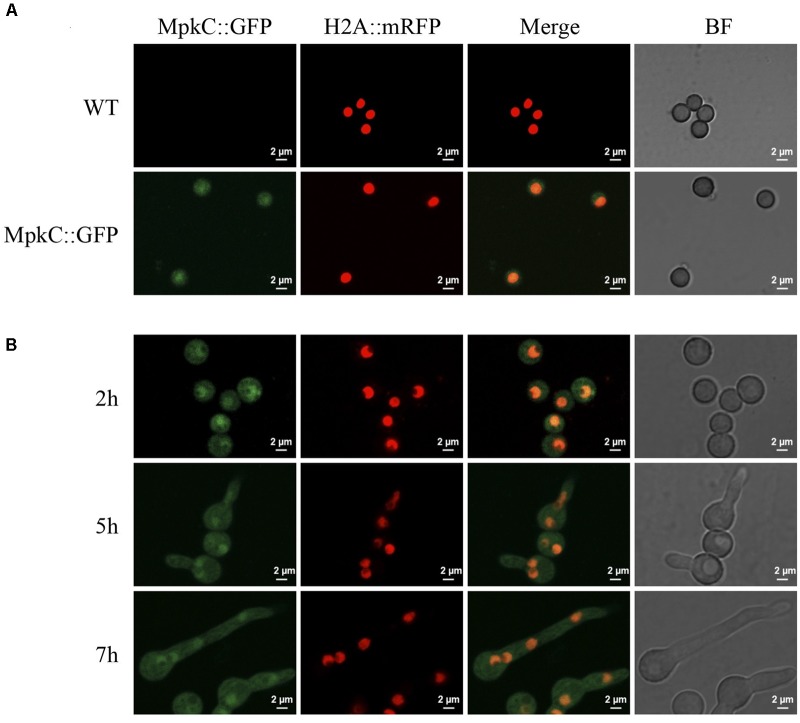
Constitutively expressed MpkC::GFP shows nuclear localization during spore germination, in the absence of stress. **(A)** Intact conidia from strains TRJ7 (WT H2A::mRFP) and TRJ13 (MpkC::GFP H2A::mRFP) were observed using confocal microscopy. **(B)** Conidia from strain TRJ13 were germinated for the indicated times and observed using confocal microscopy.

**FIGURE 5:**
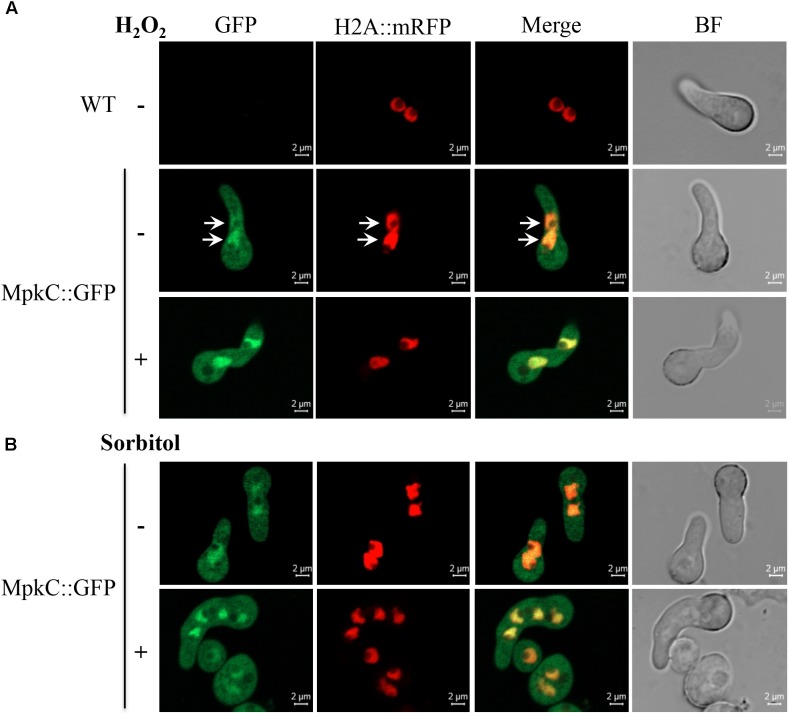
Oxidative and osmotic stresses increase the nuclear accumulation of MpkC during spore germination. **(A)** Conidia from strains TRJ7 (WT H2A::mRFP) and TRJ13 (MpkC::GFP H2A::mRFP) were germinated for 6 h in minimal medium (Top), treated or not with 10 mM H_2_O_2_ for 10 min and then observed using confocal microscopy. **(B)** Conidia from strain TRJ13 (MpkC::GFP H2A::mRFP) were germinated for 6 h in MM containing or lacking 1.2 M sorbitol and observed using confocal microscopy. Arrows in panel **(A)** point to nuclear signal.

**FIGURE 6:**
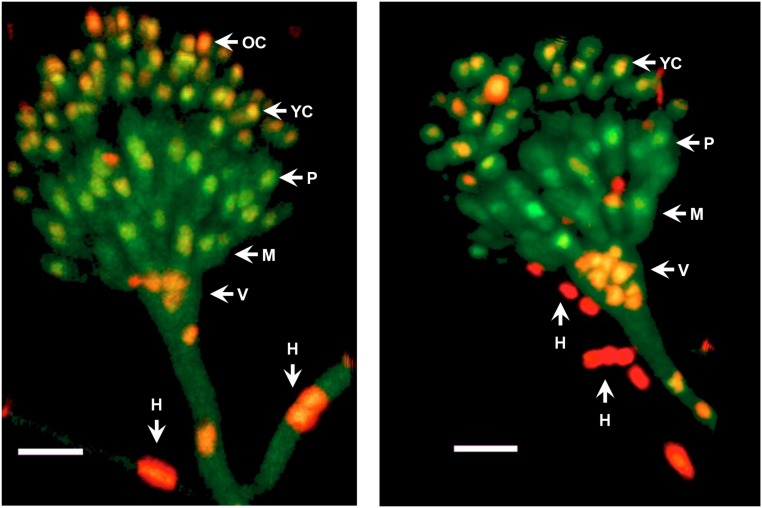
MpkC shows different nuclear localization patterns in different conidiophore cell-types. The growing edge of a colony grown for 3 days at 37°C was sectioned and observed directly by confocal microscopy. Two different conidiophores are shown. The different indicated structures are: H, hyphae; V, vesicle; M, metulae; P, phialide; YC, young conidia; and OC, older conidia. Images correspond to maximum intensity Z-stack projections. Scale bar corresponds to 5 μm.

Next, we decided to follow MpkC::GFP localization during asexual development and under stress conditions. As shown in Figure 4A, MpkC::GFP was found mostly in the cytoplasm of intact conidia, which contain a single G1-arrested nuclei. Notably, during germination an increased accumulation of MpkC::GFP was detected in nuclei, labeled with histone H2A::mCherry, during spore swelling (Figure 4B) and the establishment of polar growth and mitosis (Figures 4B, 5). Moreover, MpkC::GFP nuclear localization was further increased when germinated spores were treated with H_2_O_2_ or germinated in sorbitol medium (Figure 5). This observation was supported after measuring and comparing MpkC::GFP nuclear fluorescence intensity in non-stressed and stressed germlings (Supplementary Figure S7). These results suggest that MpkC nuclear localization is regulated during asexual development and indicate that oxidative and osmotic stress induce MpkC nuclear localization. To further explore this, we detected MpkC::GFP signal in intact conidiophores. Despite the difficulties in signal detection due to the overlap of several layers of cells, a clear red signal was observed in nuclei located in hyphae around foot cells, confirming very low levels of nuclear MpkC::GFP in hyphae (Figure 6), while different red and green intensities were observed in the different conidiophore cell-types. A clear nuclear orange color, indicative of increased MpkC nuclear localization, was observed in vesicles. Although mRFP signal decreased in metulae and phialides, green signal was clearly more intense in the center of the cell than in the periphery, suggesting a high MpkC::GFP nuclear/cytoplasmic ratio in these cells. Young conidia displayed orange and yellow signals, indicating partial nuclear localization of MpkC::GFP, while nuclear green signal decreased in older conidia, as it was also observed in isolated mature conidia (Figure 4A). This pattern of fluorescence signal suggests that while MpkC is mostly localized in the cytoplasm of hyphae, during normal conidiophore development MpkC is gradually translocated to nuclei in vesicles, metulae, and phialides, and then mostly re-localized to cytoplasm in mature conidia.

**FIGURE 7:**
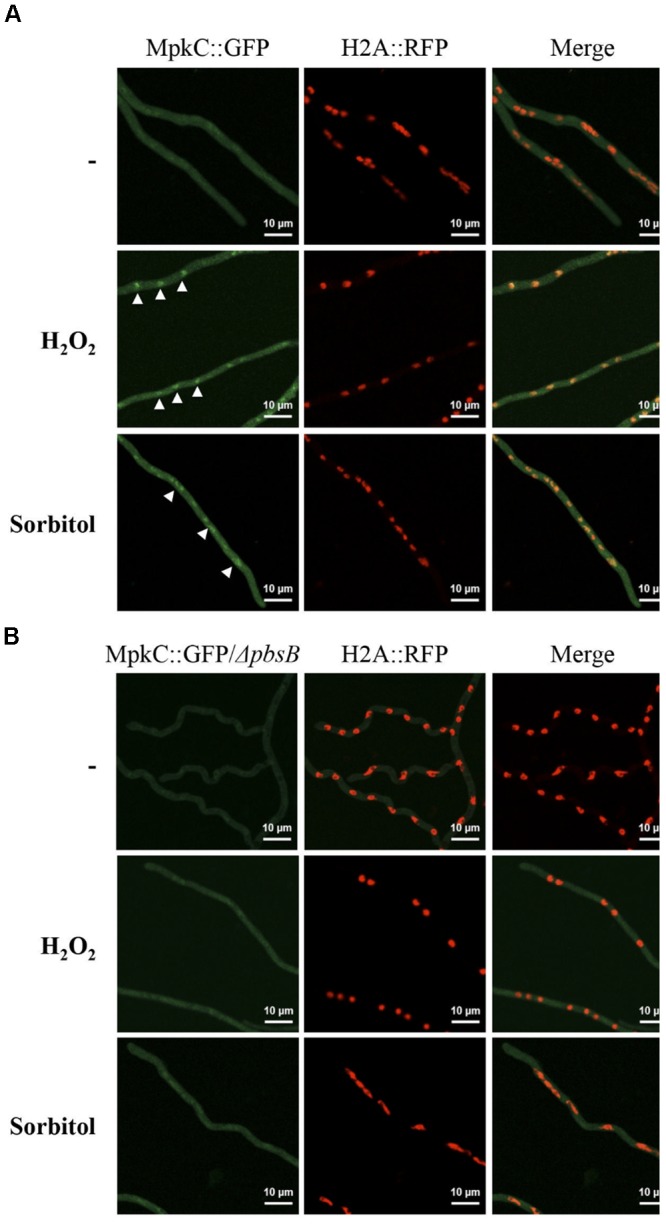
In mycelia the MAPKK PbsB is necessary for MpkC nuclear accumulation in response to oxidative and osmotic stress. **(A)** Mycelia from strain TRJ13 (MpkC::GFP H2A::mRFP) grown for 14 h in minimal medium (Top) was treated or not with 10 mM H_2_O_2_ for 20 min (Middle) or grown for 14 h on MM containing 1.2 M sorbitol and then observed using confocal microscopy. **(B)** Mycelia from strain CVG10 (*ΔpbsB* MpkC::GFP H2A::mRFP) grown for 14 h in minimal medium (Top) was treated or not with 10 mM H_2_O_2_ for 20 min (Middle) or grown for 14 h on MM containing 1.2 M sorbitol and then observed using confocal microscopy. White arrowheads in panel **(A)** point to some of the nuclei with MpkC::GFP signal.

**FIGURE 8:**
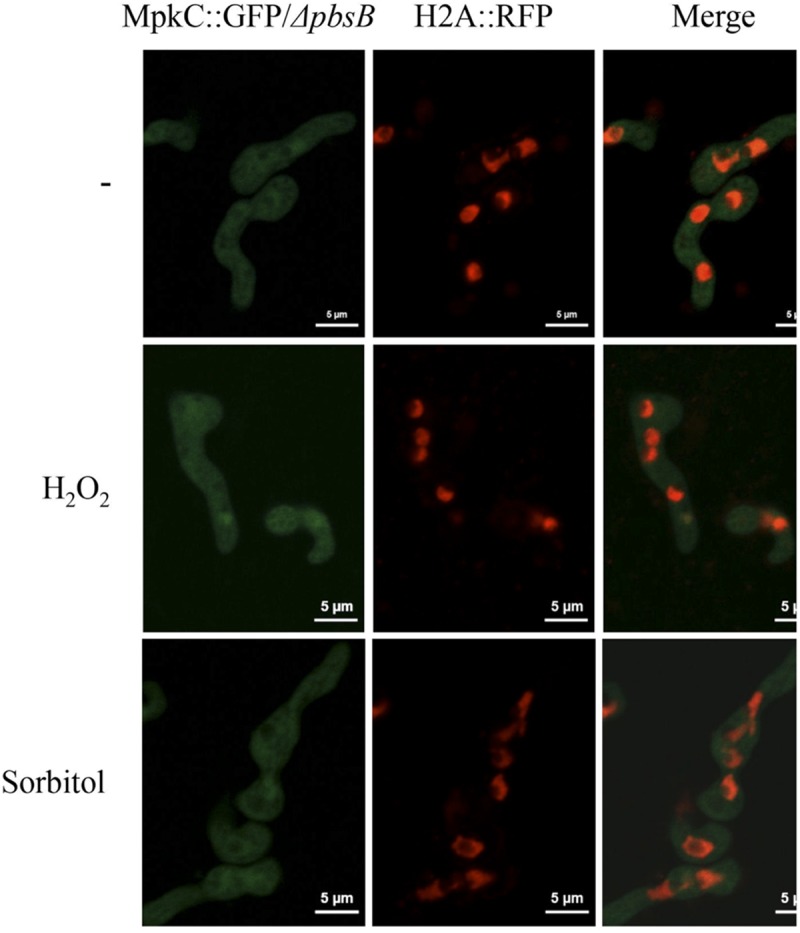
The MAPKK PbsB is necessary for MpkC nuclear accumulation during spore germination and in response to oxidative and osmotic stress. Conidia from strain CVG10 (*ΔpbsB* MpkC::GFP H2A::mRFP) were germinated for 6 h in minimal medium (Top) and treated with 10 mM H_2_O_2_ for 10 min (Middle) or germinated for 6 h in MM containing 1.2 M sorbitol and then observed using confocal microscopy.

In support of this, we found that without stress MpkC was virtually absent from nuclei in growing hyphae, while oxidative and osmotic stress induced its nuclear localization (Figure 7A). In the absence of the MAPKK PbsB, the stress-induced nuclear localization of MpkC was drastically reduced (Figure 7B). The lack of PbsB also resulted in decreased MpkC nuclear localization during conidia germination as well as during oxidative or osmotic stress (Figure 8).

In summary, these results support a model in which MpkC nuclear localization is regulated during *A. nidulans* asexual development. In this model MpkC is localized in the cytoplasm during hyphal growth, it accumulates in nuclei during conidiophore development and is largely re-localized to the cytoplasm in mature conidia. During germination, MpkC would be enriched in nuclei until hyphal growth is fully established, where MpkC becomes cytoplasmic again. Our results also show that in addition to this developmental regulation, MpkC accumulates in nuclei in response to oxidative and osmotic stress.

SakA nuclear accumulation is also regulated during development and in response to oxidative and osmotic stress. However, the developmental regulation reported for SakA (Kawasaki et al., 2002; Lara-Rojas et al., 2011; Jaimes-Arroyo et al., 2015) is opposite to what we report here for MpkC.

## Discussion

Together, our results show that SakA and MpkC have opposite as well as common functions during the *A. nidulans* life cycle and thus contribute to understand the functional relationship between these two SAPKs. Indeed, SakA and MpkC regulate conidia oxidative stress resistance in opposite ways (Figure 3) and this is consistent with their opposite nuclear localization in intact conidia, where MpkC is mostly localized in the cytoplasm (Figure 4A), and SakA is mainly localized in nuclei (Lara-Rojas et al., 2011). We ignore the mechanism by which in the absence of SakA, MpkC mediates a higher sensitivity of conidia to H_2_O_2_. Under these conditions, perhaps H_2_O_2_ induces a hyper activation of the MpkC pathway, resulting in the killing of the cell. During germination MpkC is enriched in nuclei, while SakA localize in the cytoplasm and in fact it needs to be dephosphorylated for germination to take place (Lara-Rojas et al., 2011). Moreover, SakA activity is essential while MpkC is dispensable to maintain the viability of conidia (Lara-Rojas et al., 2011; Supplementary Figure S4B). In addition to its developmental regulation, MpkC nuclear accumulation in germlings is increased by oxidative and osmotic stress treatments. MpkC roles in conidiation and stress responses can be both related to the transient regulation of cell-cycle arrest under these conditions (see further).

SakA and MpkC regulate conidiation in different ways. The lack of SakA has no major effects in conidiation, while the lack of MpkC results in an increased production of conidia, and the lack of both MAPKs and PbsB results in a drastic reduction in conidiation (Figures 1–3 and Supplementary Figure S4A). *ΔmpkC* and *ΔmpkC ΔsakA* mutant conidiophores, developed on solid media or induced by glucose or nitrogen starvation (Skromne et al., 1995) in liquid media, display normal morphology (not shown). Their respective increased and decreased conidiation, seems to result from differences in the number of conidia produced per phialide and the density of conidiophores, parameters that are difficult to evaluate. While it is clear that SakA is necessary to fully arrest mitosis in dormant conidia (Lara-Rojas et al., 2011), we propose that low kinase levels of nuclear (active) MpkC, due to low MpkC intrinsic kinase activity and/or its low expression, are necessary to delay mitosis during conidia development. In *S. pombe*, SakA ortholog Spc1/Sty1 mediates cell-cycle arrest in response to stress by phosphorylating the MAPKAP Srk1, and regulating its translocation to the nucleus (Lopez-Aviles et al., 2008). In *A. nidulans*, SakA interacts with MpkC, the Srk1 ortholog SrkA and other proteins involved in cell-cycle regulation, and also regulate SrkA nuclear localization (Jaimes-Arroyo et al., 2015). This suggests that MpkC might also regulate mitosis trough SrkA. In unicellular *S. pombe*, under poor nutrient conditions, low and high kinase levels of a single SAPK (Spc1/Sty1) suffice to regulate the advancement or the delay of mitosis, respectively (Hartmuth and Petersen, 2009; Shiozaki, 2009). In filamentous fungi cell size-mitosis control must be critical during single-cell (conidia) differentiation. Two different SAPKs might be necessary to modulate mitosis in the complex multicellular conidiophore produced by the Aspergilli, and to maintain the final dormant state of conidia. Indeed, in these fungi phialoconidiation first involves nuclei proliferation without division at the vesicle stage and later a single cell, the phialide, should undergo mitosis to produce two nuclei, one that migrates to the nascent conidia and remains arrested at G1, and the one retained by the phialide, which will undergo mitosis again in a process that in the Aspergilli is repeated many times, to produce chains of up to 120 conidia (Oliver, 1972; Mims et al., 1988; Sewall et al., 1990). In coenocytic hyphae, MpkC nuclear localization is induced by osmotic and oxidative stress, playing a minor and mostly redundant function with SakA, which explains why *mpkC* initial inactivation did not produce any clear phenotype (Jun et al., 2011).

The MAPKK PbsB mediates both, developmental and stress-induced localization of SakA and MpkC and therefore mediates SakA and MpkC phosphorylation levels. However, very low levels of MpkC nuclear signal were observed in the absence of PbsB, particularly under sorbitol treatment (Figure 8). This could be a secondary effect derived from the use of a constitute promoter to express MpkC, or it could indicate the existence of PbsB-independent mechanisms to activate MpkC. Autophosphorylation is one possibility to be explored. Although MAPKs in general do not show spontaneous autophosphorylation, such capability has been reported for p38β and in other MAPKs it could be de-repressed under specific conditions (Beenstock et al., 2014; Tesker et al., 2016).

SakA and MpkC molecular differences can easily account for their functional differences. Indeed, human CSBP2 and CSBP1, two SakA/MpkC/Hog1 homologs that are splice variants differing only in an internal 25-amino acid sequence, contrast in their ability to complement a *Δhog1* mutant and are differentially activated by salt in yeast. CSBP2 but not CSPB1 complemented a *Δhog1* yeast phenotype and yet CSPB1 was constitutively active in a PbsB2 MAPKK-dependent fashion. Notably, a CSPB1 mutant with about 3 times lower kinase activity was able to complement a *Δhog1* mutant (Kumar et al., 1995). Although both regulated by PbsB, SakA and MpkC might show differences in basal and activated kinase activity, the extent of activation by PbsB, their sensitivity to phosphatases and also in their interactions with common and different substrates.

In contrast to the different roles that SakA and MpkC play during development, they play common roles in maintaining cell-wall integrity in conidia. In *A. fumigatus* both MAPKs were shown to be individually required for resistance to osmotic, oxidative and cell-wall stress, with the simultaneous disruption of both MAPKs showing additive defects in these processes (Bruder Nascimento et al., 2016). Although we do not observe an additive effect of SakA and MpkC inactivation in oxidative or osmotic stress sensitivity, we do observe that both MAPKs contribute to cell wall integrity in conidia. In a previous report, MpkC constitutively expressed in mycelia grown for 24 h at 30**°**C, was not detected as phosphorylated, while a 10 min treatment with 0.5 M NaCl resulted in its phosphorylation (Furukawa et al., 2005). This is in agreement with our results, as we find that in mycelia MpkC is largely absent from nuclei in the absence of stress, and that both oxidative and osmotic stress treatments induce its nuclear accumulation.

## Author Contributions

JA designed the experiments, wrote the manuscript, and obtained funding. VG-B, RJ-A, OS, and FL-R performed and designed the experiments, and contributed to manuscript writing.

## Conflict of Interest Statement

The authors declare that the research was conducted in the absence of any commercial or financial relationships that could be construed as a potential conflict of interest.
