# Supplementary material for: SakA and MpkC Stress MAPKs Show Opposite and Common Functions During Stress Responses and Development in Aspergillus nidulans
Source: Front Microbiol. 2018 Oct 23;9:2518. doi: 10.3389/fmicb.2018.02518 (PMC6205964; doi:10.3389/fmicb.2018.02518)
Supplement: Supplementary file 1 [file Data_Sheet_1.PDF]

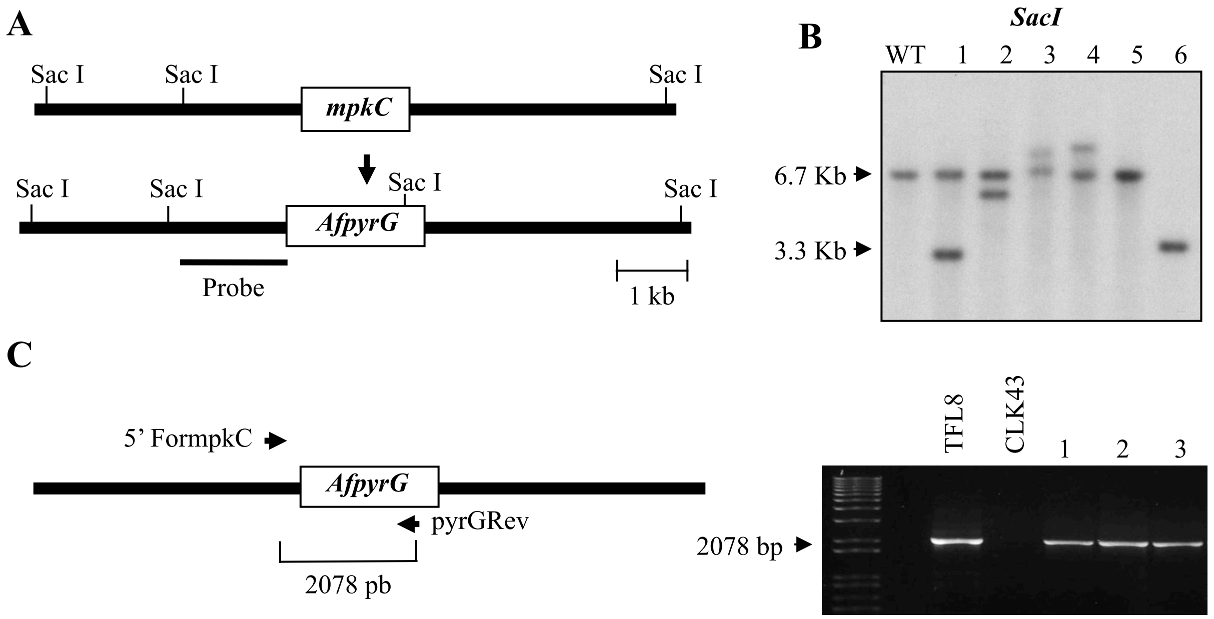

FIGURE S1. Deletion of the *mpkC* gene. **(A)** A *mpkC* deletion construct containing A*fpyrG* gene, as selective marker, was used to transform strain MH11035. **(B)** DNA from strains MH11035 (WT) and indicated PyrG^{+} transformants was digested with *SacI* and used for Southern blot analysis, using the probe indicated in **(A)**. Wild type and *ΔmpkC* patterns correspond to bands of 6.7 and 3.3 Kb, respectively; transformant 6 was renamed TFL8. **(C)** TFL8 was crossed with strain CLK43 to eliminate *nkuA* deletion (not shown) and *mpkC* deletion was confirmed by PCR. Strain 3 was renamed CFL8 and used in further experiments.

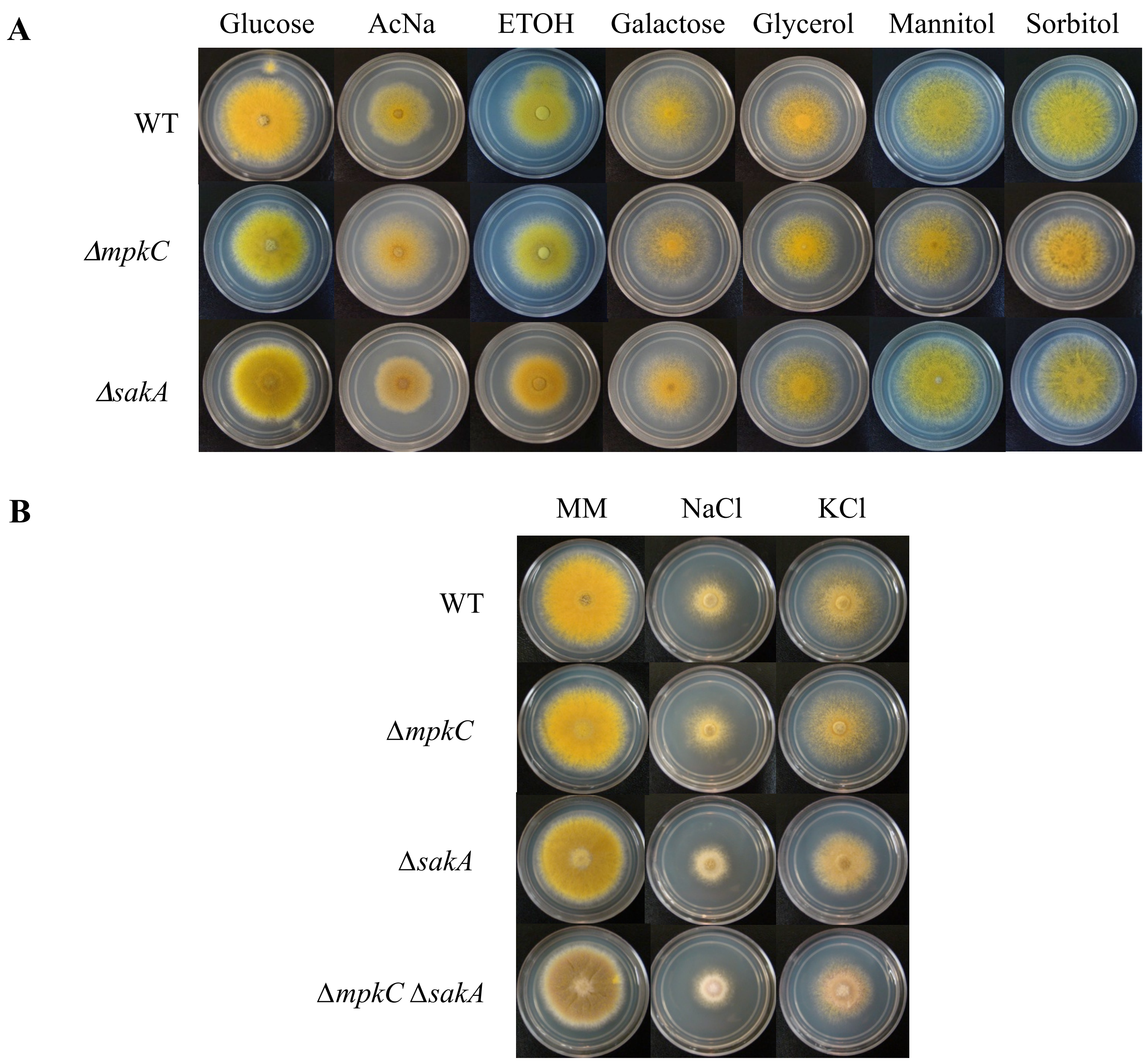

Figure S2. (A) SakA and MpkC are dispensable for alternative carbon source utilization. Conidia ($1X10^4$) from strains CLK43 (WT), CFL10 (Δ*mpkC*) and TOL1 (Δ*sakA*) were inoculated on supplemented MM plates containing either 1% glucose, 100 mM sodium acetate (AcNa), 1% ethanol (ETOH), 1% galactose, 1% glycerol, 1% mannitol or 1% sorbitol, as sole carbon sources, and incubated at 37°C during 4 days. **(B) The simultaneous inactivation of SakA and MpkC does not result in increased osmosensitivity.** Conidia ($1X10^4$) from strains CLK43 (WT), CFL8 (Δ*mpkC*), CRJ1 (Δ*sakA*) and CFL12 (Δ*mpkC* Δ*sakA*) were inoculated on supplemented MM plates containing either 1M NaCl or 0.6M KCl and incubated at 37°C for 4 days.

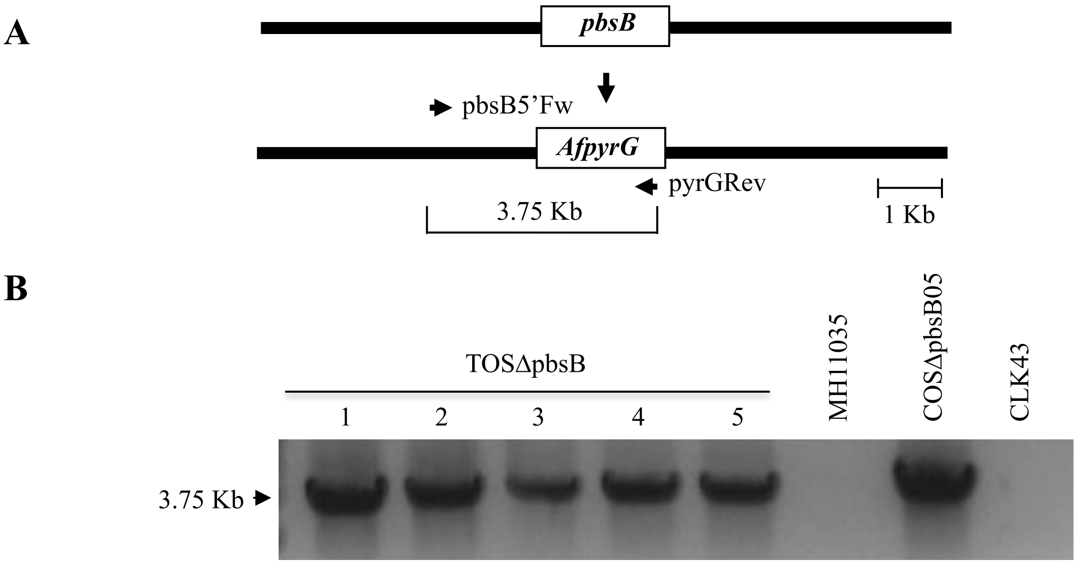

FIGURE S3. Deletion of the *pbsB* gene. (A) A *pbsB* deletion construct containing A*fpyrG* gene was used to transform strain MH11035. **(B)** PCR analysis of five pyrG^{+} transformants using primers pbsB5'Fw and pyrGRev. A 3.75 Kb band is expected only in Δ*pbsB* mutants. Strain TOSΔpbsB03 was chosen and crossed with strain CLK43 to eliminate *nkuA* deletion (not shown). Strain COSΔpbsB05 was used in additional experiments.

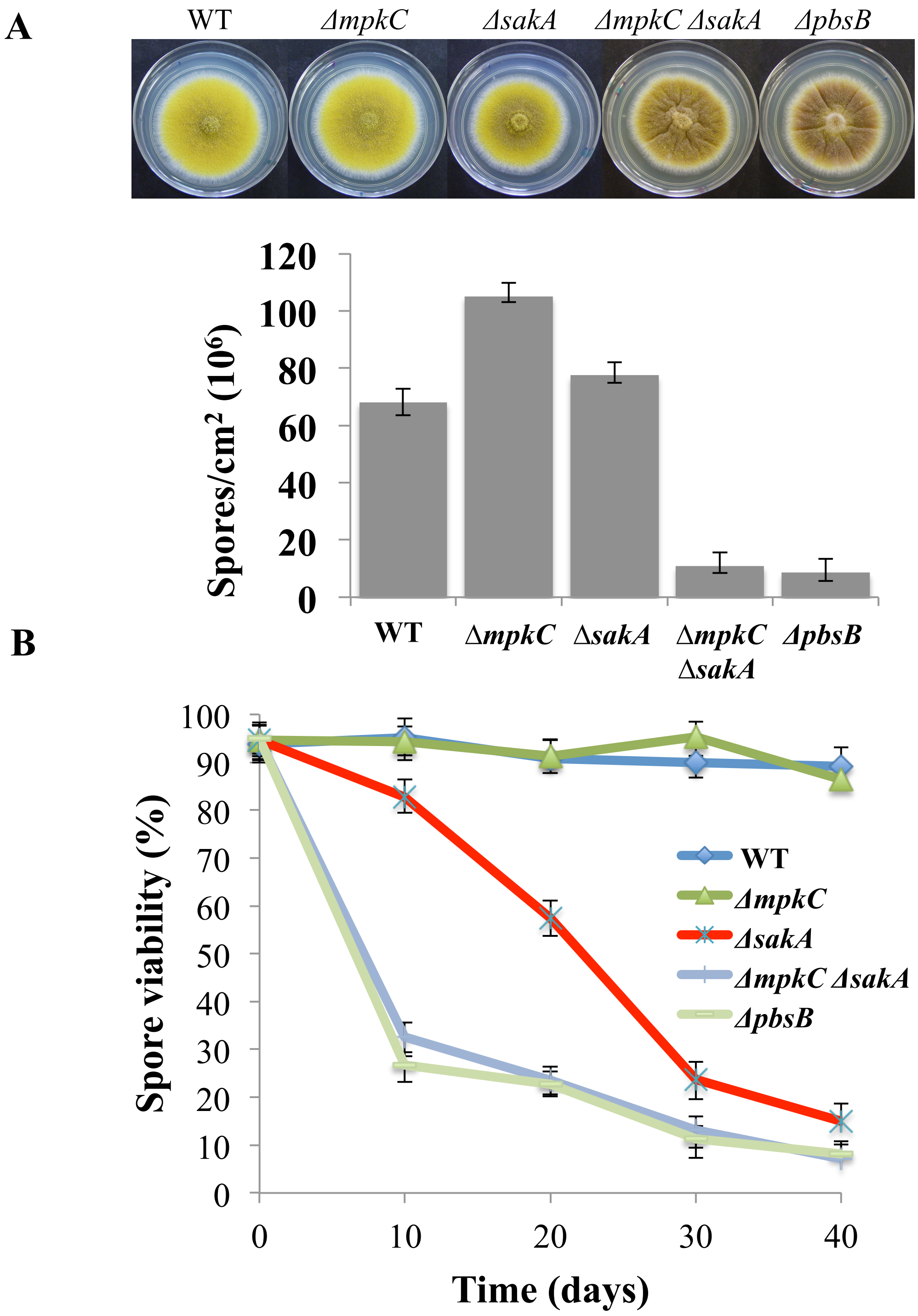

FIGURE S4. *ΔmpkC ΔsakA* and *ΔpbsB* mutants show very similar phenotypes. (A) Conidia ($1X10^4$) from strains CLK43 (WT), COS0020ΔmpkC (*ΔmpkC*), CRJ1 (*ΔsakA*), CRJ11 (*ΔmpkC ΔsakA*) and COSΔpbsB05 (*ΔpbsB*) were inoculated on supplemented MM plates and incubated at 37°C for 4 days. The top panel corresponds to strains shown in Figure 3. Total conidiospores per colony were harvested, counted and divided by the colony area. **(B)** Conidia from the same strains were collected from 5-day plates, counted and plated immediately (time 0) or maintained in water at 4°C for up to 40 days. At indicated time points, aliquots were diluted and used to inoculated supplemented MM plates and incubated at 37°C. After 2 days, the resulting colonies were counted. Data are mean values from three independent experiments; bars indicate standard deviation.

A

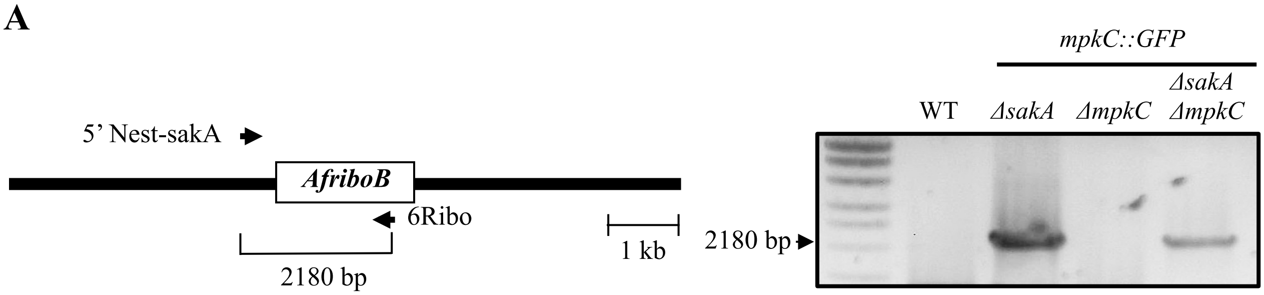

B

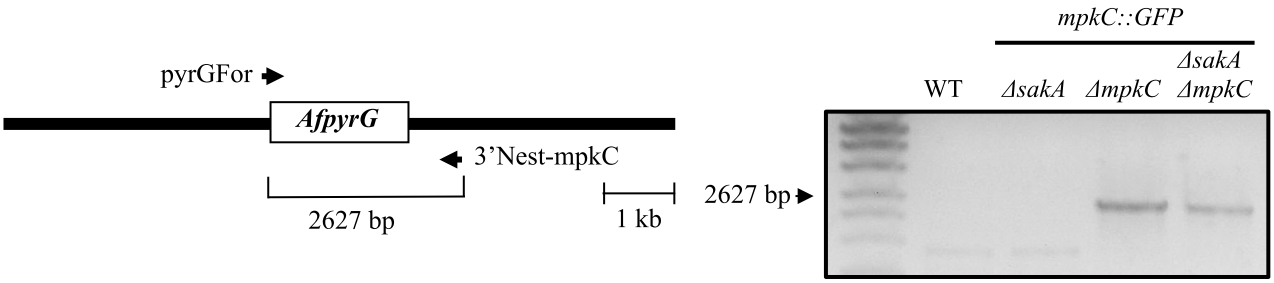

FIGURE S5. PCR confirmation of strains containing the *mpkC::GFP* allele. Genomic DNA from strains CVG16 (*ΔsakA mpkC::GFP*), CVG15 (*ΔmpkC mpkC::GFP*) and CVG17 (*ΔsakA ΔmpkC mpkC::GFP*), containing the *mpkC::GFP* allele, was used as template to amplify the PCR products indicated on the left. **(A)** *sakA* deletion was confirmed using primers 5'Nest-sakA and 6Ribo to generate a 2180 bp product, present only in *ΔsakA* mutants. (**B)** *mpkC* deletion was confirmed using primers pyrGFor and 3' Nest-mpkC to generate a 2627 bp product present only in *ΔmpkC* mutants.

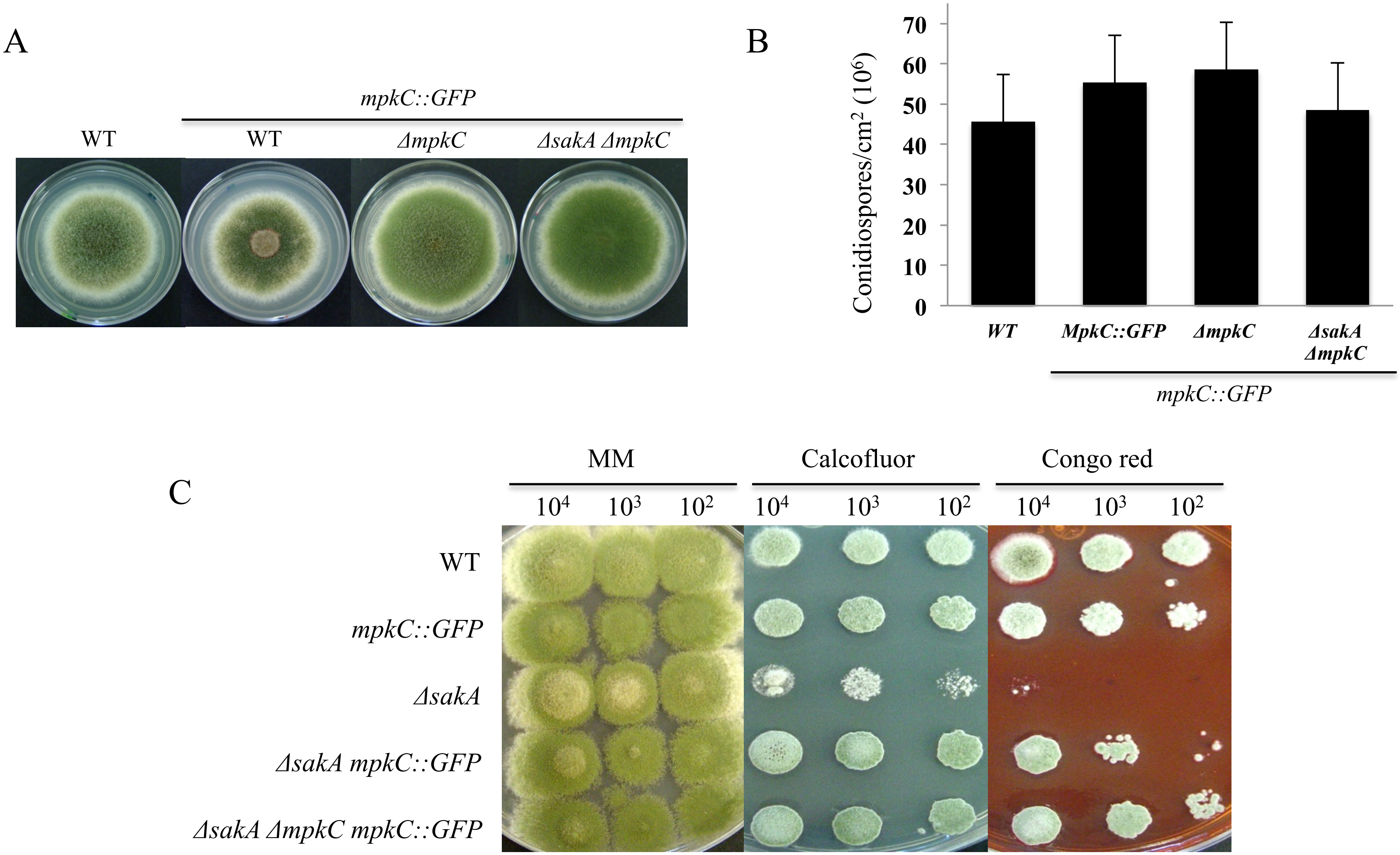

FIGURE S6. The MpkC::GFP fusion expressed constitutively is functional. (A) Conidia (1X10^4) from strains CVG13 (WT), TRJ13 (*mpkC::GFP*), CVG18 (*ΔsakA*), CVG15 (*ΔmpkC mpkC::GFP*), CVG16 (*ΔsakA mpkC::GFP*) and CVG17 (*ΔsakA ΔmpkC mpkC::GFP*) were inoculated on supplemented MM plates and incubated at 37°C for 5 days. **(B)** Total conidiospores per colony were harvested, counted, and the count divided by the colony area. Bars indicate standard deviation from three independent experiments. **(C)** The indicated number of conidia were inoculated on media containing Calcofluor (20 µg/ml) or Congo red (30 µg/ml) and incubated at 37°C for 3 days.

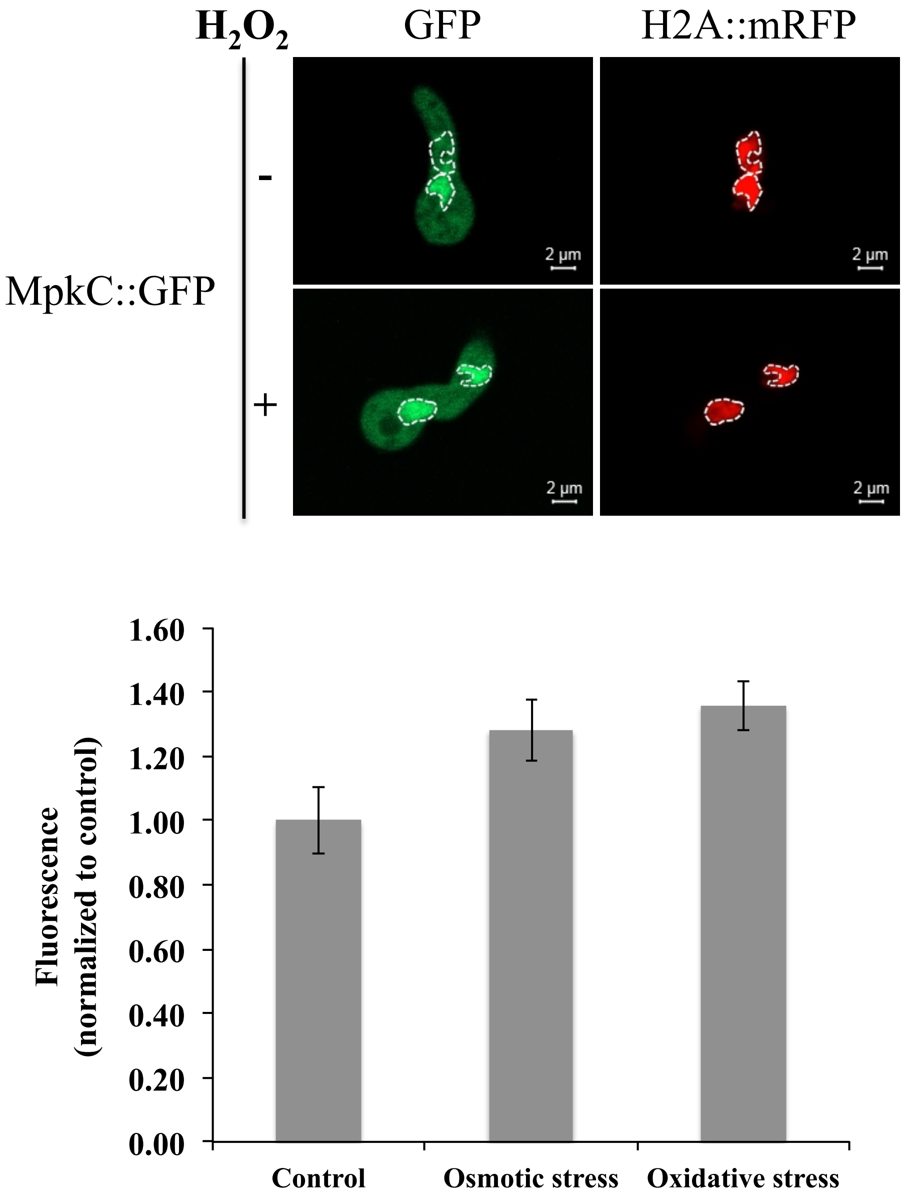

FIGURE S7. MpkC nuclear fluorescence intensity is increased in germlings subject to stress. Several images from the experiment shown in **Figure 5** were used to delineate the nuclear area as shown in the upper panel, and fluorescence intensity quantitated. Mean nuclear fluorescence intensity under stress conditions was normalized to the control (no stress). The graph shows the average of all nuclei analyzed (control n = 21, Osmotic stress n = 23 and oxidative stress n = 21). Image processing was made using Image J and ZEN 2012 software (see Materials and Methods).
