# Supplementary material for: SakA and MpkC Stress MAPKs Show Opposite and Common Functions During Stress Responses and Development in Aspergillus nidulans
Source: Front Microbiol. 2018 Oct 23;9:2518. doi: 10.3389/fmicb.2018.02518 (PMC6205964; doi:10.3389/fmicb.2018.02518)
Supplement: Supplementary file 2 [file Data_Sheet_2.docx]

**Table S1**. *Aspergillus nidulans* strains used in this study

| Strain | Genotype | Source |
| --- | --- | --- |
| CLK43 | *pabaA1 yA2 veA1* | Kawasaki *et al.,* (2002) |
| MH11035 | *pyrG89 pyroA4 riboB2* ∆*nkuA::argB veA1* | M. Hynes; Nayak *et al*., (2006) |
| FGSC A1155 | *pyrG89 pyroA4* ∆*nkuA*::*Bar veA1* | FGSC; McCluskey *et al*., (2010) |
| SRF200 | *pyrG89 ∆argB::trpC∆B pyroA4 trpC801 veA1* | Karos, M., and R. Fischer (1999) |
| TOL1 | *pabaA1 yA2 ∆argB::trpC∆B ∆sakA::argB trpC801 veA1* | Kawasaki *et al.,* (2002) |
| TFL8 | *pyrG89 pyroA4 riboB2 ∆mpkC::AfpyrG ∆nkuA::argB veA1* | This work;  MH11035 transformed with PCR construct mpkC-AfpyrG-mpkC |
| TFL11 | *pyrG89 pyroA4 riboB2 ∆sakA::AfriboB ∆mpkC::AfpyrG ∆nkuA::argB veA1* | This work; TFL∆sakA-03 transformed with PCR construct mpkC-AfpyrG-mpkC |
| TFL22 | *pyrG89 pyroA4 sakA*::*s-tag*::*AfpyrG ∆nkuA*::*Bar veA1* | Jaimes-Arroyo *et al*., (2015) |
| TFLΔsakA-03 | *pyrG89 pyroA4 riboB2 ∆sakA::AfriboB ∆nkuA::argB veA1* | This work;  MH11035 transformed with PCR construct sakA-AfriboB-sakA |
| TRJ7 | *pyrG89 pyroA4 biA::pyroA:: gpdA(p)::h2A::mrfp::biA nkuA::Bar veA1* | This work; A1155 transformed with PCR construct bio-pyroA-gpdA-h2A-mrfp-bio |
| TRJ12 | *pyrG89 pyroA4 sakA::s-tag::AfpyrG biA:: gpdA(p)::mpkC::GFP::pyroA::biA ∆nkuA::Bar veA1* | This work; TFL22 transformed with PCR construct bio-pyroA-gpdA-mpkC-GFP-bio |
| TRJ13 | *pyrG89 pyroA4 sakA::s-tag::AfpyrG biA:: gpdA(p)::mpkC::GFP::pyroA::biA gpdA(p)::h2A::mrfp::phleo ∆nkuA::Bar veA1* | This work; TRJ12 transformed with PCR construct gpdA-h2A-mrfp-phleo |
| TOS∆pbsB03 | *pyrG89 pyroA4 riboB2 ∆pbsB::AfpyrG* ∆*nkuA::argB veA1* | This work; MH11035 transformed with PCR construct pbsB-AfpyrG-pbsB |
| CFL3 | *pabaA1 yA2 pyrG89 veA1* | This work; progeny from CLK43 X SRF200 |
| CFL8 | *pabaA1 yA2 ∆mpkC::AfpyrG veA1*^a^ | This work; progeny from CFL3 X TFL8 |
| CFL10 | *pabaA1, yA2; ∆mpkC::AfpyrG; veA1*^a^ | This work; progeny from CFL3 X TFL8 |
| CFL12 | *pabaA1 yA2 ∆mpkC::AfpyrG ∆sakA::AfriboB veA1*^a,c^ | This work; progeny from TFLΔsakA-03 X CFL8 |
| CRJ1 | *pabaA1 yA2 ∆sakA::AfriboB veA1* | This work; progeny from CLK43 X TFL∆sakA-03 |
| CRJ11 | *pabaA1 yA2 ∆mpkC::AfpyrG ∆sakA::AfriboB veA1*^a,c^ | This work; progeny from TFL11 X CLK43 |
| COS∆pbsB05 | *pabaA1 yA2 ∆pbsB::AfpyrG veA1*^a^ | This work; progeny from TOS∆pbsB03 X CLK43 |
| COS0020ΔmpkC | *pabaA1 yA2 ∆mpkC::AfpyrG veA1*^a^ | This work; progeny from CFL3 X TFL8 |
| CVG13 | *biA::pyroA::gpdA(p)::h2A::mrfp::biA veA1* ^b^ | This work; progeny from TRJ7 X CLK43 |
| CVG10 | *ΔpbsB::AfpyrG biA::gpdA(p)::mpkC::GFP::pyroA::biA gpdA(p)::h2A::mrfp::phleo veA1*^ab^ | This work; progeny from COSΔpbsB05 X TRJ13 |
| CVG15 | *ΔmpkC::AfpyrG biA::gpdA(p)::mpkC::GFP::pyroA::biA veA1* ^a^ | This work; progeny from COS0020 ΔmpkC X TRJ13 |
| CVG16 | *ΔsakA::AfriboB biA::gpdA(p)::mpkC::GFP::pyroA::biA veA1* ^b,c^ | This work; progeny from CRJ1 X TRJ13 |
| CVG17 | *ΔmpkC::AfpyrG ΔsakA::Afribo biA::gpdA(p)::mpkC::*  *GFP::pyroA::biA veA1* ^a,b,c^ | This work; progeny from  CRJ11 X TRJ13 |
| CVG18 | *ΔsakA::AfriboB biA::pyroA::gpdA(p)::h2A::mrfp::biA veA1* ^b,c^ | This work; progeny from TRJ7 X CRJ1 |
| CVG19 | *ΔmpkC::AfpyrG ΔsakA::AfriboB biA::pyroA::gpdA(p)::h2A::*  *mrfp::biA veA1*^a,b,c^ | This work; progeny from CRJ11 X TRJ7 |
| CVG20 | *ΔpbsB::AfpyrG biA::pyroA::gpdA(p)::h2A::mrfp::biA veA1*^a,b^ | This work; progeny from COSΔpbsB05 X TRJ7 |

^a^It may contain *pyrG89*, ^b^it may contain *pyroA4*, ^c^it may contain *riboB2*

**Table S2**. DNA primers used in this study

| Primer | Sequence (5′ to 3′) |
| --- | --- |
| 5’For-mpkC | TACCCCAGAGCGCGATAC |
| 5’Rev-mpkC | GGTGAAGAGCATTGTTTGAGGCAGCGAAAGGAGGTGGAGT |
| 3’For-mpkC | GCATCAGTGCCTCCTCTCAGACCCAGCTCACCACAAACGC |
| 3’Rev-mpkC | GCCTTGACCTTGGCCTTG |
| pyrGforward | GCCTCAAACAATGCTCTTCACC |
| pyrGreverse | GTCTGAGAGGAGGCACTGATGC |
| 5’Nest-mpkC | TTCCGGCCAGGACACGAT |
| 3’Nest-mpkC | GGCCTTGCCCGACACTAG |
| pbsB5’Fw | TTTGGACCCTTGTATAGACGACAGC |
| pyrGpbsB5’Rv | CGCATCAGTGCCTCCTCTCAGACCCATTTACACGCCTCCGTTCA |
| pbsB3’pyrGFw | CGCATCAGTGCCTCCTCTCAGACCCATTTACACGCCTCCGTTCA |
| pbsB3’Rv | GAGCCATCGTTCATTATCAGCTT |
| RealNestedpbsB5’For | TCGGGATCCGGGAGAATGAT |
| RealNestedpbsBRev | CAATCAGCAATGCGCTGGTTC |
|  |  |
